# Relative bioavailability of fedratinib through various alternative oral administration methods in healthy adults

**DOI:** 10.1007/s00280-023-04612-w

**Published:** 2023-11-13

**Authors:** Yizhe Chen, David Wyatt, Massimo Attanasio, Mark Thomas, Michael Thomas, Bing He, Rina Nishii, Liangang Liu, Vivian Shan, Yongjun Xue, Leonidas N. Carayannopoulos, Ken Ogasawara, Gopal Krishna

**Affiliations:** 1 Bristol Myers Squibb, 556 Morris Ave, Summit, NJ 07901 USA; 2 Syneos Health, Miami, FL USA

**Keywords:** Fedratinib, Bioavailability, Drug dispersal, Pharmacokinetics, Drug administration route, Nasogastric tube

## Abstract

Fedratinib is an oral Janus kinase 2-selective inhibitor for the treatment of adult patients with intermediate-2 or high-risk myelofibrosis; however, some patients have difficulty with oral dosing. This randomized, phase 1, open-label, 2-part crossover study evaluated the relative bioavailability, safety, tolerability, taste, and palatability of fedratinib resulting from various alternative oral administration methods in healthy adults. Participants could receive fedratinib 400 mg orally as intact capsules along with a nutritional supplement; as contents of capsules dispersed in a nutritional supplement, delivered via nasogastric tube; or as a divided dose of 200 mg orally twice daily as intact capsules with a nutritional supplement. Fifty-eight participants received treatment. Total exposure to fedratinib was similar after oral administration of intact capsules or when dispersed in a nutritional supplement (area under the plasma concentration–time curve from time 0 to the time of the last quantifiable concentration geometric mean ratio [AUC_0–t_ GMR] [90% CI], 1.007 [0.929–1.092]). Total exposure to fedratinib was slightly reduced following nasogastric administration (AUC_0–t_ GMR 0.850 [0.802–0.901]) and as a divided dose (AUC_0–t_ GMR 0.836 [0.789–0.886]). No new safety signals were identified for fedratinib, and most participants found the taste and palatability acceptable when dispersed in a nutritional supplement. Overall, results suggest no clinically meaningful differences in total exposure to fedratinib between the tested oral administration methods. These findings may facilitate administration of fedratinib to patients who are intolerant of swallowing the capsule dosage form. (ClinicalTrials.gov: NCT05051553).

**Supplementary Information:**

The online version contains supplementary material available at 10.1007/s00280-023-04612-w.

## Introduction

Myelofibrosis is a myeloproliferative neoplasm associated with mutations in the cytoplasmic tyrosine kinase Janus kinase (JAK) 2, and dysregulation of the JAK/signal transducer and activator of transcription pathway [1]. Myelofibrosis is characterized by debilitating symptoms and poor survival rates [2]. Fedratinib, an oral, JAK2-selective inhibitor, is approved in the USA for the treatment of adult patients with intermediate-2 or high-risk myelofibrosis [3] and in the European Union for the treatment of splenomegaly or symptoms of disease in adult patients with myelofibrosis who are JAK-inhibitor naive or have been treated with ruxolitinib [4]. In the recent phase 3b, single-arm FREEDOM trial (NCT03755518) of 38 patients with myelofibrosis resistant/intolerant to prior ruxolitinib, fedratinib 400 mg/day reduced spleen volume (25.7% of patients achieved ≥ 35% spleen volume reduction at the end of cycle 6; 62.9% of patients had a best overall response of ≥ 35% spleen volume reduction at any time) and myelofibrosis symptom burden (44.4% of patients achieved ≥ 50% reduction in total symptom score at end of cycle 6) [5].

The current fedratinib 400 mg dosing is given as 4 × 100-mg, 21.4–22.0 mm (size 0) hard capsules, and is recommended to be taken with food [4]. However, for those patients who have difficulty swallowing solid-form medications [6], solid-form dosing may lead to reduced treatment adherence or treatment modifications by patients, potentially affecting safety and efficacy [7, 8]. Patients with myelofibrosis also experience a perception of early satiety and abdominal pain/discomfort resulting from spleen enlargement [9], which may contribute to an aversion to orally administered medication. Those who cannot take medications by mouth may require a nasogastric tube; however, alternative administration routes can affect medication pharmacokinetics (PKs) and bioavailability, and alter the benefit/risk profile of medications for patients [10, 11]. Previous studies have found that fedratinib shows a biphasic disposition and linear, time-invariant PKs [12], with a 3 h median (range, 2–4 h) time to peak concentration of the current fedratinib 400 mg dosing in patients with myelofibrosis [3]. The results of two phase 1 studies in healthy volunteers demonstrated that food has minimal impact on the bioavailability of fedratinib at 100 mg and 500 mg doses. Both studies showed a slight increase in maximum observed plasma concentration (C_max_), time to C_max_ (T_max_), area under the plasma concentration–time curve (AUC) when fedratinib was administered directly after food compared with a 10-h fasted condition; however, increases in exposure were not clinically significant (< 25%) and terminal elimination half-life (t_½_) remained unaffected by fed and fasted conditions. Fedratinib tolerability was improved when taken following a high-fat breakfast [13].

Nausea is also a potential side effect of fedratinib. A total of 39.5% of patients in the FREEDOM study and 62% of patients in the JAKARTA trial experienced nausea [2, 5]. The improvement of gastrointestinal effects in the FREEDOM study versus JAKARTA was likely due to the administration of concomitant antiemetic medications, such as ondansetron, before fedratinib administration. These medications have been shown to reduce fedratinib-related nausea and vomiting without affecting the PKs of fedratinib [16], and are administered to all participants in postmarketing clinical pharmacology studies [17–19]. Additionally, the administration of fedratinib alongside a nutritional supplement has been found also to help alleviate nausea symptoms [3, 4].

In an effort to facilitate treatment of patients with myelofibrosis who have difficulty with the current fedratinib solid dosage form, this study evaluated administration of fedratinib dispersed in a nutritional supplement (either orally or via nasogastric tube) compared with intact capsules. In addition, to reduce the pill burden for patients who have difficulty taking all 4 × 100-mg capsules of fedratinib in a single dose, study part 2 investigated the impact of splitting the fedratinib intact capsule dose into 2 × 100-mg capsules administered twice daily (BID) on the total exposure to fedratinib compared with 4 × 100-mg capsules administered once daily (QD) alongside a nutritional supplement. The primary objective of this study (NCT05051553) was to evaluate the relative bioavailability of fedratinib 400 mg when administered orally as the contents of capsules dispersed in a nutritional supplement, the contents of capsules dispersed in a nutritional supplement via nasogastric tube, or as intact capsules with a nutritional supplement as a divided dose (200 mg BID) in comparison to intact capsules with a nutritional supplement in healthy adults.

The secondary objective was to evaluate the safety and tolerability of fedratinib administered by these methods in healthy adults. This study also had an exploratory objective of determining the taste and palatability of fedratinib when capsule contents were dispersed in a nutritional supplement.

## Methods

### Ethics approval

This phase 1 (NCT05051553), open-label, 2-part crossover study in healthy adult participants was conducted in compliance with the International Council on Harmonisation of Technical Requirements for Registration of Pharmaceuticals for Human Use/Good Clinical Practice and applicable regulatory requirements. The protocol complied with the Declaration of Helsinki as well as applicable guidelines of the USA, the country where the study was conducted. The protocol was submitted to an independent review board (Advarra, Columbia, MD, USA) for review and written approval. This study was run by Syneos Health (Miami, FL, USA). Written informed consent was obtained from all participants at screening, prior to the conduct of any study-related procedures.

### Study population

Healthy adults aged 18–65 years, with a body mass index 18–33 kg/m^2^, were eligible to be enrolled in either part of the study. Participants were confirmed to be healthy based on normal/clinically acceptable vital signs, laboratory results, and electrocardiograms at screening. People of childbearing potential were neither pregnant nor lactating, and all participants were required to use highly effective contraceptive measures until 30 days after the last dose of the study drug. Participants with a history of Wernicke’s encephalopathy, thiamine deficiency, hypersensitivity to ondansetron or any components of study drug, or contraindications for insertion of a nasogastric tube were excluded from the study.

### Study design and treatment

This study comprised 2 parts in a randomized, multiple-sequence design. The study parts were planned to run in any order, or in parallel. Participants could only join 1 part of the study. Each study part was composed of a screening, a treatment phase, and a follow-up phone call (approximately 4 days [± 2 days] after discharge).

**Fig. 1:**
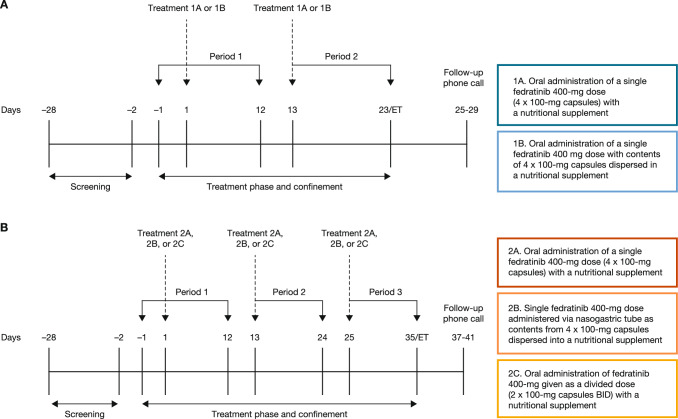
Study design for study part 1 (**A**) and part 2 (**B**). *BID* twice daily; *ET* early termination

Study part 1 was a 2-period, 2-sequence, open-label, crossover design (Fig. 1A). Treatment 1A (reference) consisted of fedratinib 400 mg (4 × 100-mg capsules) administered orally along with approximately 180 mL of a commercially available nutritional supplement (ie, Ensure Plus). Treatment 1B (test) consisted of fedratinib 400 mg dispersed in a nutritional supplement and administered orally. Participants were randomized to receive treatment in one of the following sequences: 1A–1B or 1B–1A. All participants underwent a supervised overnight fast of ≥ 10 h the night before fedratinib dosing. No food or beverages (except water and any nutritional supplement administered with the treatment) were allowed for at least 4 h after dosing.

Study part 2 was a 3-period, 6-sequence, open-label, crossover design (Fig. 1B). Treatment 2A (reference) consisted of fedratinib 400 mg (4 × 100-mg capsules) administered orally alongside a nutritional supplement. Treatment 2B (test) consisted of fedratinib 400 mg dispersed in the nutritional supplement (180 mL, Ensure Plus), administered via nasogastric tube, and flushed with 60 mL of sterile water. Treatment 2C (test) consisted of fedratinib 400 mg as a divided dose (2 × 100-mg capsules BID) administered alongside the nutritional supplement. Participants were randomized to receive treatment in one of the following sequences: 2A–2B–2C, 2A–2C–2B, 2B–2A–2C, 2B–2C–2A, 2C–2A–2B, or 2C–2B–2A. As in study part 1, all participants underwent a supervised overnight fast of ≥ 10 h the night before dosing. In the case of an evening (second) dose in the divided dosing plan of treatment 2C, participants were directed to pre-fast at least 2 h before fedratinib dose, and 4 h after fedratinib dose, with water restricted from approximately 2 h before and 1 h after dosing, excluding water given with the treatment.

Approximately 1 h before each fedratinib administration, ondansetron oral tablet (film coated) 8 mg was given orally to reduce the potential for fedratinib-related nausea and vomiting [16]. There was a washout period of at least 12 days between each dose of fedratinib. The duration from screening through the follow-up phone call was approximately 10 weeks. Participants resided at the clinical site from days -1 to 23 (study part 1), or days -1 to 35 (study part 2). If a participant discontinued from the study for any reason, an early termination visit was performed, with only the safety assessments scheduled for the day of discharge taking place. Additionally, each participant who discontinued also received a follow-up phone call 4 (± 2) days after completion of the early termination visit.

### Pharmacokinetic sampling, bioanalysis, and variables

For study part 1, and part 2 treatments 2A and 2B, blood samples were collected pre-dose (0 h) and at 0.5, 1, 1.5, 2, 3, 4, 6, 8, 12, 24, 48, 72, 120, 168, 192, and 240 h post-dose. For treatment 2C, blood samples were collected pre-dose (0 h) and at 0.5, 1, 1.5, 2, 3, 4, 6, 8, 12, 12.5, 13, 13.5, 14, 15, 16, 18, 24, 48, 72, 120, 168, 192, and 240 h post-dose of the first dose. The plasma concentration of fedratinib was measured using a validated liquid chromatography-tandem mass spectrometry assay with a lower limit of quantification of 1.00 ng/mL [12, 20].

Plasma PK parameters were calculated using noncompartmental methods by a validated PK analysis program (Phoenix^®^ WinNonlin^®^, v8.3.4 [Certara USA, Inc., Princeton, NJ, USA]) using actual times. The PK parameters determined for fedratinib C_max_, T_max_, AUC from time 0 to the time of the last quantifiable concentration (AUC_0–t_), AUC from time 0 to infinity (AUC_0–∞_), and t_½_. Additionally for treatment 2C, C_max_ and T_max_ were estimated after both the first dose (C_max_ and T_max_) and the second dose (C_max2_ and T_max2_). AUC_0–t_ and AUC_0–∞_ were estimated from time 0 (the first dose) through 240 h after the first dose. AUC parameters were calculated using the linear-log trapezoidal method.

### Safety assessment

The safety population included all participants who received at least 1 dose of fedratinib. Participants were monitored for adverse events (AEs) during the study, including assessments for clinical symptoms, biochemical, hematologic, and urinalysis laboratory results, physical examinations, 12-lead electrocardiograms, and vital signs. AEs were assessed for seriousness, severity, and relationship to the drug. AEs were assigned to the last study treatment administered at the time of onset, starting during or after the administration of the first dose. AEs that occurred after discharge were assigned to the last study treatment received for up to 30 days after the last dose of fedratinib.

### Taste and palatability assessment

As an exploratory endpoint, participants in part 1 were asked to answer a questionnaire regarding taste and palatability on days 1 and 13 of the study. The questionnaire assessed participant perceptions of bitterness, astringency, and sandiness/grittiness at 2, 5, and 15 min after administration, other taste attributes, and impulse to chew or swallow.

### Statistical considerations

The sample size for this study was based on consideration of the precision in the comparison of PK parameters, represented by the 90% confidence interval (CI) of the geometric mean ratios of C_max_ and AUC of fedratinib. The evaluable PK population, used for PK summary and statistical analyses, was defined as all participants who received at least 1 dose of fedratinib and had at least 1 evaluable PK parameter. Only participants with valid PK data were included in the summary statistics and statistical analysis.

To compare the PK parameters and estimated relative bioavailability of fedratinib following different administration methods in each study part (treatment 1B to 1A, and treatment 2B and 2C to 2A), a linear mixed-effect model with treatment, period, and sequence as fixed effects and participant nested within sequence as a random effect was fitted to the natural log-transformed PK parameters (C_max_, AUC_0–t_, and AUC_0–∞_) for use in estimation of effects and construction of CIs.

In study parts 1 and 2, point estimates and 90% CI for the difference between test and reference treatments was exponentiated, and the ratio of geometric means (test/reference) and associated 90% CI was presented on the original scale. For comparison of treatment 2C to 2A in study part 2, the relative bioavailability assessment was based on only AUC. For T_max_, the difference between test and reference was assessed for significance using Wilcoxon signed-rank test. Kenward–Rogers degrees of freedom were specified in the linear mixed-effect model. The Hodges–Lehmann estimate and its 90% CI were calculated for median difference between treatments.

All statistical analyses were conducted using SAS v9.4 (SAS Institute Inc., Cary, NC, USA).

## Results

### Participants and disposition

A total of 161 participants were enrolled, and 58 were randomized (36.0%) into the study and included in the PK and safety analyses. Of the 58 participants, 28 were included in study part 1 and 30 were included in study part 2 (Table 1). In total, 4 participants discontinued from the study, including 3 due to AEs (part 1, 2; part 2, 1); 1 participant withdrew from the study in part 1 (Table S1). Participant demographics and baseline characteristics were similar between the treatment groups and study parts (Table 1). For study parts 1 and 2, the mean age of participants was 38.5 and 41.7 years, respectively, and most participants were male (53.6% and 66.7% in parts 1 and 2, respectively; Table 1).

**Table 1:** Population demographics and baseline characteristics

	Part 1 *n* = 28	Part 2 *n* = 30
Age		
Mean, years (Min–max)	38.5 (21–62)	41.7 (20–63)
Height		
Mean, centimeters (Min–max)	165.4 (151–179.5)	167.1 (151–181)
Weight		
Mean, kilograms (Min–max)	74.9 (50.4–96.7)	76.1 (48–97.5)
BMI, kg/m^2^		
Mean (Min–max)	27.4 (20.8–32.2)	27.1 (20.9–31.8)
Sex, *n* (%)		
Female Male	13 (46.4) 15 (53.6)	10 (33.3) 20 (66.7)
Race, *n* (%)		
Black or African American White	10 (35.7) 18 (64.3)	4 (13.3) 26 (86.7)
Ethnicity, *n* (%)		
Hispanic or Latino Not Hispanic or Latino	28 (100) –	28 (93.3) 2 (6.7)

*BMI* body mass index, *max* maximum, *min* minimum

**Table 2:** Summary statistics for pharmacokinetic parameters in study parts 1 and 2

	Treatment 1A (*n* = 27)	Treatment 1B (*n* = 26)	Treatment 2A (*n* = 30)	Treatment 2B (*n* = 30)	Treatment 2C (*n* = 30)
C_max_, ng/mL (Geo. %CV)	1319 (42.0)	1433 (40.0)	1388 (35.0)	1246 (37.0)	641^a^ (41.0)
C_max2_, ng/mL (Geo. %CV)	–	–	–	–	487.3^b^ (44.0)
AUC_0–t,_ ng·h/mL (Geo. %CV)	20,040 (41.0)	20,515 (46.0)	22,638 (38.0)	19,245 (35.0)	18928^c^ (40.0)
AUC_0–∞,_ ng·h/mL (Geo. %CV)	22,121 (39.0)	22,685 (43.0)	25,034 (38.0)	21,600 (35.0)	21275^c^ (40.0)
T_max_, h (range)	2.1 (1.5–5.9)	2.0 (0.6–3.0)	2.9 (1.4–6.1)	1.9 (1.0–3.0)	1.9^a^ (1.4–6.0)
T_max2_, h (range)	–	–	–	–	3.9^b^ (1.0–6.0)
t_½_, h (SD)	103.0 (30.0)	98.7 (28.2)	96.8 (27.6)	106.0 (31.4)	96.7^d^ (26.2)

*AUC*_*0–t*_ area under the plasma concentration–time curve from time 0 to last quantifiable concentration, *AUC*_*0–∞*_ area under the plasma concentration–time curve from time 0 to infinity, *BID* twice daily, *C*_*max*_ maximum observed plasma concentration, *C*_*max2*_ maximum observed plasma concentration after second dose is presented, *CV* coefficient of variation, *Geo* geometric, *SD* standard deviation, *T*_*max*_ time to maximum observed plasma concentration, *T*_*max2*_ time to maximum observed plasma concentration after second dose is presented, *t*_*½*_ terminal elimination half-life

Pharmacokinetic parameters are presented as geometric mean (CV%) except T_max_ where the median (minimum, maximum) values are presented and t_½,_ where the mean (SD) is presented

1A, oral administration of a single fedratinib 400-mg dose (4 × 100-mg capsules) with a nutritional supplement; 1B, oral administration of a single fedratinib 400-mg dose with contents of 4 × 100-mg capsules dispersed into a nutritional supplement; 2A, oral administration of a single fedratinib 400-mg dose (4 × 100-mg capsules) with a nutritional supplement; 2B, single fedratinib 400-mg dose administered via nasogastric tube as contents from 4 × 100-mg capsules dispersed into a nutritional supplement; 2C, oral administration of fedratinib 400 mg given as a divided dose (2 × 100-mg capsules BID) with a nutritional supplement

^a^C_max_ and T_max_ after the first dose are presented

^b^C_max_ and T_max_ after second dose are presented

^c^AUC_0–t_ and AUC_0–∞_ were estimated from time 0 (the first dose) through 240 h after the first dose

^d^t_½_ after the second dose

## Relative bioavailability of fedratinib and PK parameters by oral administration method

### Study part 1

**Fig. 2:**
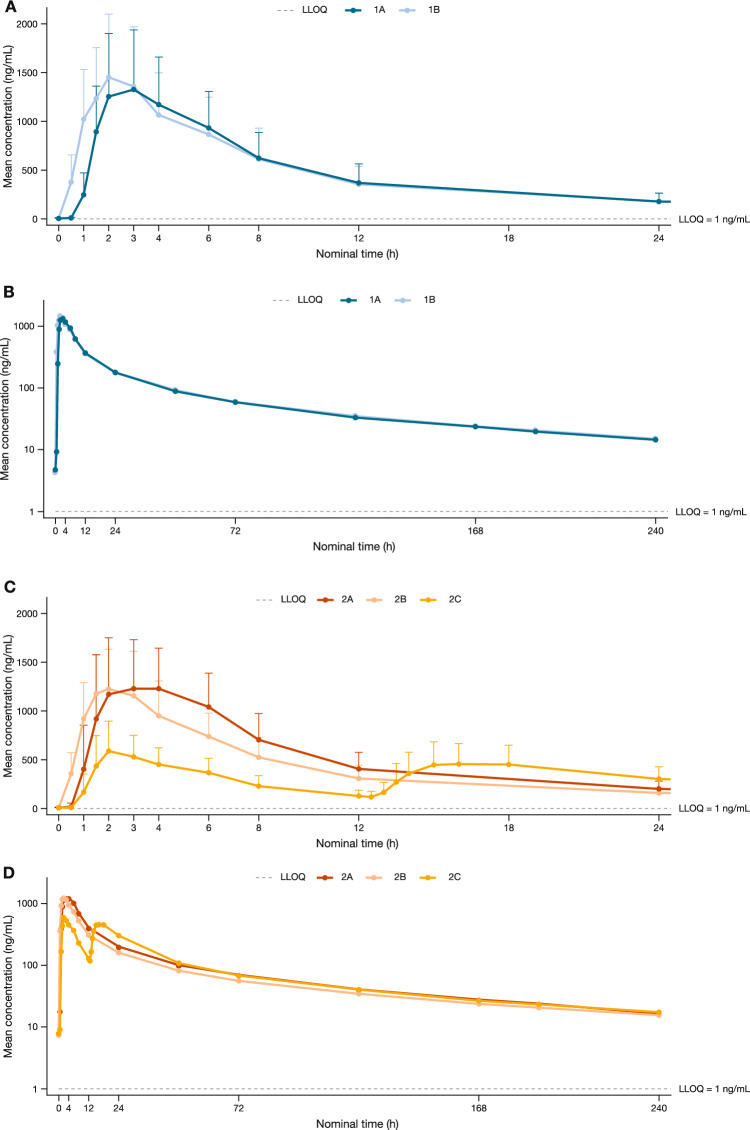
Fedratinib plasma concentration profile over time. Part 1 mean (+ SD) plasma concentration of fedratinib over time in linear (**A**) and semilogarithmic scales (**B**), and part 2 mean (+ SD) plasma concentration of fedratinib over time in linear (**C**) and semilogarithmic scales (**D**). On the linear profiles, points show mean + SD. BID, twice daily; LLOQ, lower limit of quantification; SD, standard deviation. 1A, oral administration of a single fedratinib 400-mg dose (4 × 100-mg capsules) with a nutritional supplement; 1B, oral administration of a single fedratinib 400-mg dose with contents of 4 × 100-mg capsules dispersed into a nutritional supplement; 2A, oral administration of a single fedratinib 400-mg dose (4 × 100-mg capsules) with a nutritional supplement; 2B, single fedratinib 400-mg dose administered via nasogastric tube as contents from 4 × 100-mg capsules dispersed into a nutritional supplement; 2C, oral administration of fedratinib 400 mg given as a divided dose (2 × 100-mg capsules BID) with a nutritional supplement

Absorption of fedratinib occurred rapidly for both treatment 1A (intact capsules with nutritional supplement) and 1B (contents of capsules dispersed in nutritional supplement). In treatment 1A, the median (range) T_max_ occurred at 2.1 (1.5–5.9) hours, compared with 2.0 (0.6–3.0) hours for treatment 1B (Table 2). After reaching C_max_, the decline in plasma concentration was gradual and prolonged for both treatment 1A and 1B (Fig. 2A and B), as indicated by long t_½_ (mean [standard deviation (SD)]: 103.0 [30.0] and 98.7 [28.2] hours for treatments 1A and 1B, respectively; Table 2). The ratio of geometric means (90% CI) for PK parameters between treatments 1B and 1A showed no apparent difference in exposure, with 1.007 (0.929–1.092) for AUC_0–t_, 1.009 (0.939–1.085) for AUC_0–∞_, and 1.059 (0.969–1.157) for C_max_ (Table 3).

**Table 3:** Study part 1 – Statistical analysis of pharmacokinetic parameters

	Geometric mean^a^		Geometric mean ratio^b,c^ (90% CI)
	Treatment 1A (*n* = 27)	Treatment 1B (*n* = 26)	Treatment 1B vs 1A
C_max_, ng/mL	1350.874	1430.527	1.059 (0.969–1.157)
AUC_0–t,_ ng∙h/mL	20,377.821	20,523.247	1.007 (0.929–1.092)
AUC_0–∞,_ ng∙h/mL	22,499.843	22,700.447	1.009 (0.939–1.085)
T_max_, h	2.574	1.997	–0.492 (–0.984 to –0.026)

*AUC*_*0–t*_ area under the plasma concentration–time curve from time 0 to last quantifiable concentration, *AUC*_*0–∞*_ area under the plasma concentration–time curve from time 0 to infinity, *CI* confidence interval, *C*_*max*_ maximum observed plasma concentration, *T*_*max*_ time to maximum observed plasma concentration

1A, oral administration of a single fedratinib 400-mg dose (4 × 100-mg capsules) with a nutritional supplement; 1B, oral administration of a single fedratinib 400-mg dose with contents of 4 × 100-mg capsules dispersed into a nutritional supplement

^a^Median for T_max_ are presented

^b^Geometric mean ratio values have been calculated from a linear mixed-effect model

^c^Median difference and 90% CI for T_max_ are presented

**Table 4:** Study part 2 – Statistical analysis of pharmacokinetic parameters

	Geometric mean^a^			Geometric mean ratio^b,c^ (90% CI)	
	Treatment 2A (*n* = 30)	Treatment 2B (*n* = 30)	Treatment 2C^**d**^ (*n* = 30)	Treatment 2B vs 2A	Treatment 2C^d^ vs 2A
C_max_, ng/mL	1387.829	1246.192	641.398	0.898 (0.837–0.963)	0.481 (0.447–0.516)
AUC_0–t,_ ng·h/mL	22,637.955	19,245.412	18,928.186	0.850 (0.802–0.901)	0.836 (0.789–0.886)
AUC_0–∞,_ ng·h/mL	25,034.449	21,599.540	21,275.302	0.863 (0.816–0.912)	0.850 (0.804–0.899)
T_max_, h	2.669	1.873	2.264	–0.958 (–1.267 to –0.517)	–0.476 (–0.584 to –0.216)

*AUC*_*0–t*_ area under the plasma concentration–time curve from time 0 to last quantifiable concentration, *AUC*_*0–∞*_ area under the plasma concentration–time curve from time 0 to infinity, *BID* twice daily, *CI* confidence interval, *C*_*max*_ maximum observed plasma concentration, *T*_*max*_ time to maximum observed plasma concentration

2A, oral administration of a single fedratinib 400-mg dose (4 × 100-mg capsules) with a nutritional supplement; 2B, single fedratinib 400-mg dose administered via nasogastric tube as contents from 4 × 100-mg capsules dispersed into a nutritional supplement; 2C, oral administration of fedratinib 400 mg given as a divided dose (2 × 100-mg capsules BID) with a nutritional supplement

^a^Median for T_max_ are presented

^b^Geometric mean ratio values have been calculated from a linear mixed-effect model

^c^Median difference and 90% CI for T_max_ are presented

^d^C_max_ and T_max_ after first dose are presented

Interparticipant variability across treatments was moderate, with coefficient of variation (CV) ranging from 39 to 46% for C_max_, AUC_0–t_, and AUC_0–∞_ across treatments 1A and 1B (Table 2).

### Study part 2

#### *Treatment 2B (dispersed capsules, nasogastric tube) versus Treatment 2A (intact capsules)*

The median (range) T_max_ occurred rapidly in both treatments, at 2.9 (1.4–6.1) hours for treatment 2A and at 1.9 (1.0–3.0) hours for treatment 2B (Table 2). The decline in plasma fedratinib concentration after C_max_ was gradual and prolonged with both treatments 2A and 2B (Fig. 2C and D), with a mean (SD) t_½_ of 96.8 (27.6) and 106.0 (31.4) hours, respectively. Overall, fedratinib exposure was slightly reduced for treatment 2B compared with 2A. The ratio of geometric means (90% CI) for PK parameters between treatment 2B and 2A was 0.850 (0.802–0.901) for AUC_0–t_, 0.863 (0.816–0.912) for AUC_0–∞_, and 0.898 (0.837–0.963) for C_max_ (Table 5).

Interparticipant variability across treatments was moderate, with CV ranging from 35 to 38% for C_max_, AUC_0–t_, and AUC_0–∞_ across treatments 2A and 2B (Table 2).

#### *Treatment 2C (divided dose, 2* × *100-mg intact capsules BID) versus treatment 2A (4* × *100-mg intact capsules)*

Absorption of fedratinib was rapid for treatment 2C and treatment 2A, with median (range) T_max_ occurring at 1.9 (1.4–6.0) and 2.9 (1.4–6.1) hours, respectively, after first doses. After the second dose of treatment 2C, the median (range) T_max2_ was 3.9 (1.0–6.0) hours (Table 2). The geometric mean C_max_ for treatment 2C after the first dose (641.0 ng/mL) was approximately half of the C_max_ of treatment 2A (1388.0 ng/mL), as expected. After reaching C_max_, the decline of plasma concentration was gradual and prolonged (Fig. 2C and D), with the mean (SD) t_½_ of fedratinib being similar between treatment 2A (96.8 [27.6] hours) and treatment 2C (96.7 [26.2] hours). Overall, exposure to fedratinib was reduced for treatment 2C compared with treatment 2A. The ratio of geometric means (90% CI) for PK parameters between treatment 2C and treatment 2A was 0.836 (0.789–0.886) for AUC_0–t_ and 0.850 (0.804–0.899) for AUC_0–∞_ (Table 4).

Interparticipant variability for treatment 2C was moderate, with CV of 40% to 41% for C_max_, AUC_0–t_, and AUC_0–∞_ (Table 2).

### Safety

Overall, 38/58 (65.5%) of participants in the safety population had at least 1 AE. The number and proportion of participants who reported AEs was similar among all treatment groups. In study part 1, 11/27 (40.7%) participants who received treatment 1A and 10/27 (37.0%) participants who received treatment 1B reported at least 1 AE (Table S2). In study part 2, 9/30 (30.0%) participants who received treatment 2A, 9/30 (30.0%) participants who received treatment 2B, and 12/30 (40.0%) participants who received treatment 2C reported at least 1 AE (Table S2). In the full safety population (n = 58) the most common AEs were constipation (12.1%), diarrhea (10.3%), and somnolence (10.3%). Most AEs were mild in severity (4 moderate AEs were reported by 3 participants in study part 2), suspected to be related to the study drug, and resolved without any medication. While no deaths or serious/severe AEs were reported during the study, 3 participants discontinued due to AEs, with 2 AEs suspected to be related to the study drug (gamma-glutamyl transferase and alanine transaminase increases, which resolved within 2 months without medication; influenza-like illness, which resolved within 2 weeks of onset; and ear pain, which resolved within 2 days of onset).

### Taste and palatability

In part 1A (fedratinib 400 mg [4 × 100-mg capsules] administered orally alongside a nutritional supplement), 100% of participants with recorded assessments reported the bitterness, astringency, and sandiness to be acceptable at all time points, and most participants (55.6%) had an impulse to swallow. In part 1B (fedratinib 400 mg dispersed in a nutritional supplement and administered orally), all participants with recorded assessments found the bitterness, astringency, and sandiness associated with drug administration to be acceptable after initial drug administration, and 77.8% of participants had an impulse to swallow. After 15 min, 96.3% of participants found the astringency and sandiness to be acceptable, while bitterness acceptability was 81.5%. Overall, the reporting of unacceptable taste and palatability was higher when the contents of the capsule were dispersed in nutritional supplement (treatment 1B) compared with swallowing an intact capsule (treatment 1A).

## Discussion

It is important to investigate alternative routes of oral administration to assist with patient comfort and treatment adherence [8]; however, changes in oral administration methods may alter the PK properties of the compound, thus affecting bioavailability [7, 8]. This study evaluated impact of various alternative oral dosing options on fedratinib bioavailability.

In part 1 of this study, exposure to fedratinib was shown to be similar following oral administration of intact capsules (treatment 1A), or oral administration of the contents of capsules dispersed in a nutritional supplement (treatment 1B). T_max_ occurred slightly sooner with treatment 1B, possibly as no time was required for capsule disintegration, and the fine particles in the capsule contents may have dissolved quickly. In part 2, exposure to fedratinib was slightly reduced following administration via a nasogastric tube (treatment 2B) or as a divided dose (treatment 2C) compared with the one-time orally administered dose (treatment 2A), though no clinically meaningful differences were observed. Reduced exposure with nasogastric tube administration compared with oral formulation has been observed in multiple compounds with distinct PK properties, although the extent varies and exact reasons have not been well understood [10, 21–24]. For example, small differences in C_max_ and AUC values between intact capsules and nasogastric administration have been observed for gefitinib, a tyrosine kinase inhibitor, with no clinically significant differences in bioavailability and PK parameters found overall [22].

In this study, reduced exposure following administration via a nasogastric tube was unlikely to be caused by drug instability or inadequate flushing, as in vitro studies demonstrated near-complete drug recovery from the nasogastric tube and the compound remained stable up to 1 h in suspension (data on file). Reduced exposure was also unlikely to be due to misplacement of the nasogastric tube, as placement was confirmed through X-ray imaging (data on file). The differences in exposure to fedratinib between treatments 2B, 2C, and 2A were likely to be due to interparticipant variability. Furthermore, the 90% CI for the difference in exposure to fedratinib between treatments 2B and 2A fell within the 0.8–1.25 range conventionally defined as having no clinical effect [25–28]. While the lower boundary of the 90% CI of the difference in AUC_0–t_ between 2C and 2A only fell slightly lower than 0.8 (0.789), the other PK results in this study would suggest that there were no clinically meaningful differences between 2C and 2A.

Within study part 2, T_max2_ was slightly longer than T_max_ after the first dose in treatment 2C. This was likely due to a shorter fasting period, approximately 3 h, compared with the overnight fasting in treatments 2B and 2A and before the first dose of treatment 2C, as food has been shown to affect the T_max_ of fedratinib without affecting fedratinib exposure [13].

There is a general lack of published PK data for kinase inhibitors through alternative oral administration routes, which may be due to the general poor aqueous solubility of small molecular tyrosine kinase inhibitors [29]. Exploratory PK analyses of patients who received baricitinib, a JAK inhibitor indicated for use in some patients with moderately to severely active rheumatoid arthritis [30], as a solution of crushed tablets via a nasogastric tube were consistent with PK results previously reported for baricitinib in healthy adults and in patients with rheumatoid arthritis [31]. To our knowledge, this is the first study evaluating the effect of alternative administration methods on the PK parameters of a JAK inhibitor for myelofibrosis. While ruxolitinib can be administered through a nasogastric tube and the prescribing information contains specific instructions for the suspension, dilution, and timing of administration [32], the effect of this route of administration on drug exposure has not been demonstrated [32]. The prescribing information for pacritinib, like fedratinib, states that capsules should not be opened, broken, or chewed [33], but no studies of the effect on pacritinib exposure appear to have been published. Thus, no data about other JAK inhibitors are available for comparison with the fedratinib data from this study.

Few oral cancer drugs report enteral tube administration alternatives, and fewer still have instructions of nasogastric administration [34]. Vandetanib, gefitinib, and osimertinib, all tyrosine kinase inhibitors used in various cancer therapies, state in their prescribing information that they can be administered through a nasogastric tube, and each have specific instructions for the suspension, dilution, and timing of administration [35–37], though only gefitinib appears to have published bioavailability and safety results of different administration methods [22, 38]

Overall, no new safety signals were identified compared with the FREEDOM study and other previous studies, including any treatment-emergent serious AEs [2, 5, 19, 39]. The number and proportion of participants experiencing AEs was similar among all treatment groups. Most of the AEs suspected to be related to fedratinib were mild in intensity and resolved without medication, though 3 individuals were discontinued from the study due to AEs.

Lastly, most participants in study part 1 found the taste and palatability of fedratinib administration to be acceptable for both treatment 1A and 1B. The taste and palatability of alternative administration methods is an important consideration as taste, texture, and smell may affect overall treatment adherence [6–8]

Some potential limitations of the study include that the participants in study part 2C, oral administration of fedratinib 400 mg given as a divided dose (2 × 100-mg capsules BID) with a nutritional supplement, were unable to have the same fasting period for both doses due to practical reasons, potentially affecting the results as described above. Finally, not all participants in study part 1A had taste and palatability reported at all time points; therefore, the assessment of taste and palatability could not be interpreted with rigor.

## Conclusion

Overall, the results of this study suggest that there is no clinically meaningful difference in exposure to fedratinib 400 mg/day after nasogastric tube administration or oral administration as a divided dose (2 × 100-mg capsules BID) compared with standard capsule administration (4 × 100-mg/day). These findings provide an alternative means of administering fedratinib to patients with myelofibrosis who are intolerant of swallowing capsule dosage forms.

### Supplementary Information

Below is the link to the electronic supplementary material.

Supplementary file1 (DOCX 55 KB)

## Data Availability

The data that support the findings of this study are available on request from Bristol Myers Squibb. The data are not publicly available due to privacy or ethical restrictions. The Bristol Myers Squibb policy on data sharing may be found at https://www.bms.com/researchers-and-partners/independent-research/data-sharing-request-process.html.
