# Supplementary material for: Relative bioavailability of fedratinib through various alternative oral administration methods in healthy adults
Source: Cancer Chemother Pharmacol. 2023 Nov 13;93(4):307–17. doi: 10.1007/s00280-023-04612-w (PMC10950972; doi:10.1007/s00280-023-04612-w)
Supplement: Supplementary file 1 — Supplementary file1 (DOCX 55 KB) [file 280_2023_4612_MOESM1_ESM.docx]

# Supplementary materials

Table S1 Participant disposition and exposure

| **Study part 1 treatments** | | | | | | | |
| --- | --- | --- | --- | --- | --- | --- | --- |
| **Participant status, *n* (%)** | **Seq AB**  **(*n* = 14)** | **Seq BA**  **(*n* = 14)** | **Total**  **(*N* = 28)** |  |  |  |  |
| Completed the study | 12 (85.7) | 13 (92.9) | 25 (89.3) |  |  |  |  |
| Did not complete the study | 2 (14.3) | 1 (7.1) | 3 (10.7) |  |  |  |  |
| Discontinued due to AE | 1 (7.1) | 1 (7.1) | 2 (7.1) |  |  |  |  |
| Discontinued due to withdrawal by participant | 1 (7.1) | 0 | 1 (3.6) |  |  |  |  |
| **Study part 2 treatments** | | | | | | | |
| **Participant status, *n* (%)** | **Seq ABC**  **(*n* = 5)** | **Seq ACB**  **(*n* = 5)** | **Seq BAC**  **(*n* = 5)** | **Seq BCA**  **(*n* = 5)** | **Seq CAB**  **(*n* = 5)** | **Seq CBA**  **(*n* = 5)** | **Total**  **(*N* = 30)** |
| Completed the study | 5 (100.0) | 5 (100.0) | 5 (100.0) | 5 (100.0) | 5 (100.0) | 4 (80.0) | 29 (96.7) |
| Did not complete the study | 0 | 0 | 0 | 0 | 0 | 1 (20.0) | 1 (3.3) |
| Discontinued due to AE | 0 | 0 | 0 | 0 | 0 | 1 (20.0) | 1 (3.3) |

*AE* adverse event, *BID* twice daily, *Seq* sequence

1A, oral administration of a single fedratinib 400-mg dose (4 x 100-mg capsules) with a nutritional supplement; 1B, oral administration of a single fedratinib 400-mg dose, with contents of 4 x 100-mg capsules dispersed in a nutritional supplement; 2A, oral administration of a single fedratinib 400-mg dose (4 x 100-mg capsules) with a nutritional supplement; 2B, single fedratinib 400-mg dose administered via nasogastric tube as contents from 4 x 100-mg capsules dispersed into a nutritional supplement; 2C, oral administration of fedratinib 400 mg given as a divided dose (2 x 100-mg capsules BID) with a nutritional supplement.

Table S2 AEs by intensity

| **Participants with AE**  ***n* (%)** | **1A**  ***n* = 27** | **1B**  ***n* = 27** | **2A**  ***n* = 30** | **2B**  ***n* = 30** | **2C**  ***n* = 30** |
| --- | --- | --- | --- | --- | --- |
| Mild | 11 (40.7) | 10 (37.0) | 9 (30.0) | 8 (26.7) | 10 (33.3) |
| Moderate | 0 | 0 | 1 (3.3) | 1 (3.3) | 2 (6.7) |
| Severe | 0 | 0 | 0 | 0 | 0 |
| Total | 11 (40.7) | 10 (37.0) | 9 (30.0) | 9 (30.0) | 12 (40.0) |

*AE* adverse event, *BID* twice daily

1A, oral administration of a single fedratinib 400-mg dose (4 x 100-mg capsules) with a nutritional supplement; 1B, oral administration of a single fedratinib 400-mg dose with contents of 4 x 100-mg capsules dispersed into a nutritional supplement; 2A, oral administration of a single fedratinib 400-mg dose (4 x 100-mg capsules) with a nutritional supplement; 2B, single fedratinib 400-mg dose administered via nasogastric tube as contents from 4 x 100-mg capsules dispersed into a nutritional supplement; 2C, oral administration of fedratinib 400 mg given as a divided dose (2 x 100-mg capsules BID) with a nutritional supplement
